# Changes in proBDNF and Mature BDNF Levels After Medium-Intensity Functional Motor Rehabilitation Program in Patients with Parkinson’s Disease

**DOI:** 10.3390/ijms26083616

**Published:** 2025-04-11

**Authors:** Joanna Cholewa, Marta Nowacka-Chmielewska, Agnieszka Gorzkowska, Andrzej Malecki, Anetta Lasek-Bal, Jaroslaw Cholewa

**Affiliations:** 1 Institute of Sport Sciences, Academy of Physical Education in Katowice, 40-065 Katowice, Poland; a.cholewa@awf.katowice.pl; 2 Laboratory of Molecular Biology, Institute of Physiotherapy and Health Sciences, Academy of Physical Education in Katowice, 40-065 Katowice, Poland; m.nowacka@awf.katowice.pl (M.N.-C.); a.malecki@awf.katowice.pl (A.M.); 3 Department of Neurology, School of Health Sciences, Medical University of Silesia in Katowice, 40-635 Katowice, Poland; agorzkowska@sum.edu.pl (A.G.); abal@sum.edu.pl (A.L.-B.)

**Keywords:** Parkinson’s disease, proBDNF, BDNF, physical rehabilitation, exercise

## Abstract

Physical rehabilitation complements the treatment of Parkinson’s disease (PD). The applied physical exercises are effective in PD by promoting activity-dependent neuroplasticity. The main aim of this study was to assess the effect of a 16-week moderate-intensity functional physical rehabilitation program (FPR) on the concentration of mature brain-derived neurotrophic factor (BDNF) and its precursor (proBDNF) in blood serum and the severity of symptoms and quality of life in people with PD. People with PD (Hoehn and Yahr stage 3) were randomly assigned to the experimental (FPR) and control (CG) groups. FPR participated in movement training to improve functional mobility, motor coordination, and balance. Pre- and post-intervention assessments included serum levels of proBDNF, mature BDNF, MDS-UPDRS sub-scales, and the PDQ-39 quality of life measured. In the FPR group, a statistically significant increase in serum proBDNF levels by 39.42% (*p* = 0.006) was observed, as well as an improvement in motor and non-motor aspects of daily functioning, motor complications, and overall quality of life. No statistically significant changes in BDNF levels were observed. The results indicate that moderately intensive FPR enhances neurotrophic mechanisms, primarily through regulating proBDNF and improving motor functions and quality of life in patients with PD. The results underline the potential of targeted rehabilitation programs to increase neuroplasticity and improve clinical outcomes in PD.

## 1. Introduction

The chronic neurodegenerative condition Parkinson’s disease (PD) presents significant complexity, with treatment typically relying on levodopa and dopamine agonists aimed at symptom management [1]. Consequently, non-pharmacological approaches are increasingly incorporated into interdisciplinary therapeutic plans for PD. Recent studies underscore the role of targeted motor rehabilitation, with evidence showing that structured resistance training can enhance physical function and quality of life [1,2,3]. Furthermore, aerobic exercise has shown benefits for motor function, while balance training, recommended as part of a comprehensive regimen, can also yield functional benefits [4,5]. Cycling, Nordic walking, dancing, and Tai Chi are advocated for improving mobility and physical fitness in patients with PD [6,7]. Research has also highlighted that supervised high-intensity interval training (HIIT) improves cardiorespiratory fitness and motor symptoms safely over the short term [8].

Despite numerous studies, there are still no clear recommendations for physical activity (PA) that would consider the intensity, specificity, difficulty, and complexity of exercises, which are essential parameters driving neuroplasticity [9,10].

The beneficial effects of exercise on clinical outcomes are thought to involve neurotrophic mechanisms, notably the synthesis of brain-derived neurotrophic factor (BDNF), which is vital for neurogenesis, synaptic plasticity, and neuronal survival [11,12,13]. In contrast, the precursor form, proBDNF, may contribute to neurodegeneration, with studies indicating its involvement in apoptosis and the inhibition of neural stem cell functions [14,15]. However, the research of Petzinger et al. [16] showed that physical exercise increases proBDNF, which may promote the regeneration of dopaminergic pathways. On this basis, it can be concluded that proBDNF requires further research and a search for interventions that will explain its importance [16].

PA has been shown to influence BDNF levels, which may be therapeutically beneficial for PD symptom management. Structured exercise, such as treadmill training, has been shown to elevate plasma BDNF in patients with PD, correlating with improvements in gait, balance, and motor symptoms [17]. Lower plasma exosomal BDNF levels have also been associated with postural instability and gait disturbance, indicating that PA may help alleviate these symptoms by boosting BDNF [18]. Animal studies further support this, showing that exercise-induced BDNF increases can protect dopaminergic neurons, potentially slowing neurodegeneration in PD [19].

Exercise intensity is also relevant; higher-intensity exercise elicits more substantial BDNF release than lower-intensity activities. However, these elevations are transient, emphasizing the importance of regular exercise for sustained BDNF levels [20]. The release of BDNF from the brain, facilitated by PA, is linked to maintaining neuronal health and cognitive function, particularly in aging and neurodegenerative contexts [21]. These findings suggest that structured PA could serve as a valuable adjunct in PD management by enhancing BDNF levels, thereby supporting neuroprotection and motor function.

Considering that neurotrophin signaling pathways are involved in various neurodevelopmental and neurophysiological processes [22], it appears that BDNF plays a crucial role among factors implicated in the pathogenesis of mental and neurodegenerative disorders. Based on numerous studies indicating that decreased serum or plasma levels of BDNF in depressed patients recovered to normal levels after antidepressant treatment [23], it was proposed that circulating BDNF levels may be a biomarker of depression [24]. Furthermore, an experimental study by Klein et al. [25] supports the hypothesis that BDNF concentrations in the blood may reflect brain BDNF levels.

In post-mortem studies of patients with PD, reduced brain BDNF levels and mRNA expression have been well documented. For example, the levels of BDNF mRNA and protein were found to be reduced in the substantia nigra of patients with PD [26]. Recently, clinical studies have shown alterations in BDNF levels in the serum of patients with PD [27,28,29]. In the serum of newly diagnosed patients with PD, low levels of BDNF were detected, and its concentration was positively correlated with a longer time of the disease, the severity of the PD symptoms, and more advanced stages of the disease [30].

The neuroprotective effects of forced exercise were demonstrated in an animal model of Parkinsonism induced by the administration of dopaminergic neurotoxins [31,32]. Notably, these effects have included enhanced protein levels of BDNF. This may be relevant to patients with PD [33].

PA has been shown to influence BDNF concentrations in individuals with PD. Physical exercise can enhance BDNF levels, which may have therapeutic implications for managing PD symptoms. Studies have demonstrated that exercise, mainly structured programs like treadmill training, can increase plasma BDNF levels. Belchior et al. [17] reported that an 8-week treadmill training regimen significantly improved PD patients’ gait, balance, and plasma BDNF levels, suggesting a direct correlation between PA and BDNF concentration [17]. Similarly, Chung et al. [18] reported that lower levels of plasma exosomal BDNF were associated with postural instability and gait disturbance in patients with PD, indicating that PA activity might mitigate these symptoms by enhancing BDNF levels [18].

Moreover, the neuroprotective effects of BDNF are further supported by findings from animal studies. Palasz et al. [19] highlighted that treadmill training in a chronic mouse model of PD increased midbrain BDNF levels, contributing to the protection of dopaminergic neurons. This aligns with the notion that PA elevates BDNF levels and promotes neuroprotection, potentially slowing the progression of neurodegeneration [34]. The intensity of exercise also plays a role in BDNF release. According to research by Schmidt-Kassow et al. [20], high-intensity exercise leads to more pronounced increases in serum BDNF compared to low-intensity activities, with BDNF levels returning to baseline shortly after exercise cessation [20]. This transient nature of BDNF elevation underscores the importance of regular PA to maintain elevated BDNF levels over time.

The relationship between PA and BDNF is supported by evidence showing that exercise enhances BDNF release from the brain, as Seifert et al. [35] noted. This release is believed to be crucial for maintaining neuronal health and cognitive function, particularly in aging populations and those with neurodegenerative diseases [21]. In summary, PA positively affects BDNF concentrations in individuals with PD, promoting neuroprotection and potentially alleviating motor symptoms. The evidence suggests that structured exercise programs can be an effective adjunctive therapy in managing PD, enhancing physical and cognitive functions through increased BDNF levels.

This study aimed to assess the effect of a 16-week moderate-intensity functional physical rehabilitation program (FPR) on the concentration of brain-derived neurotrophic factor (BDNF) and its precursor (proBDNF) in blood serum and the severity of symptoms and quality of life in people with PD.

## 2. Results

Before the analysis, both groups were compared regarding age, disease duration, MDS-UPDRS score, and PDQ-39 test. The studies showed no statistically significant differences between the groups (Table 1).

Considering this study’s aim, the proBDNF and BDNF results and the MDS-UPDRS and PDQ-39 test results are presented in separate tables (Table 2).

After assessing changes in proBDNF concentration, statistically significant differences were found after the analysis of variance, considering the factor of participation in FPR and time (F = 6.555, *p* < 0.015). Analyzing the results of post hoc tests, statistically significant differences were found in the FPR group after the intervention. In the study period, after 16 weeks of exercise, a significant (*p* = 0.006) increase of 39.42% in the level of proBDNF was observed in the FPR group. There were also differences between the FPR and CG groups after the intervention period (20.96%).

**Figure 1:**
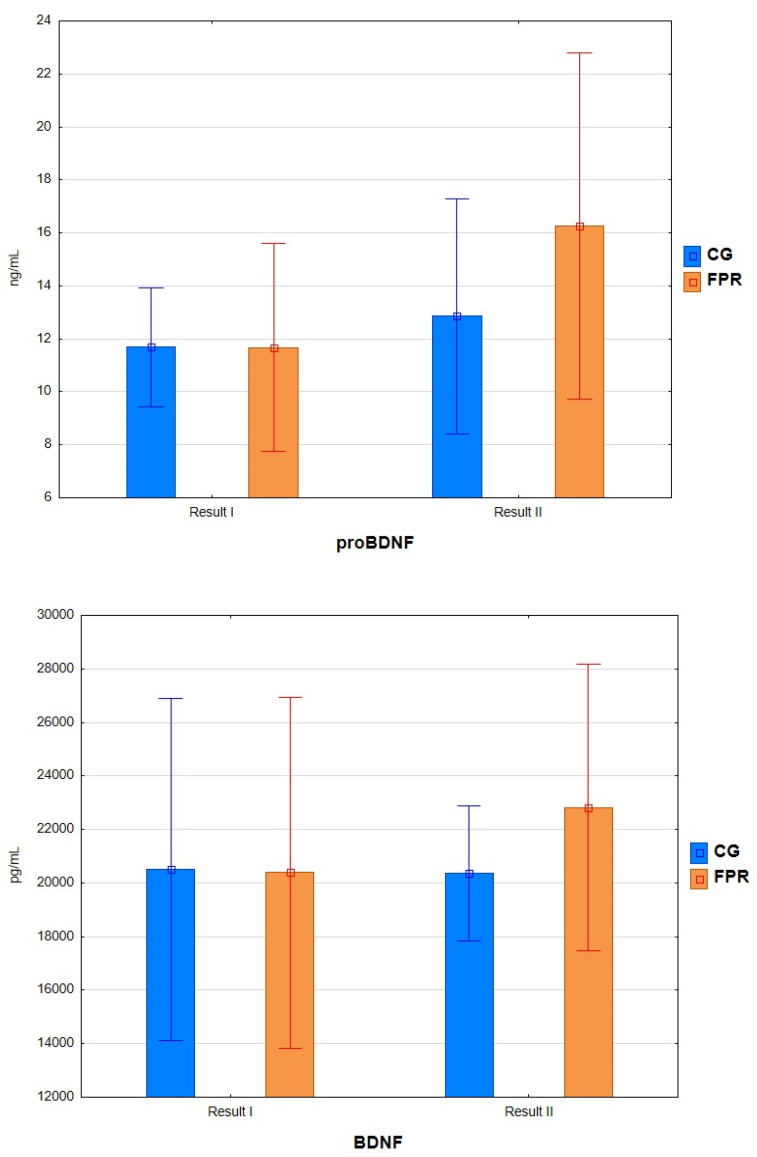
Concentration of the tested parameters before and after the experiment.

Due to the lack of assumptions about the normality of the distribution, the comparison of BDNF measurements was made using the Wilcoxon signed-rank test. The calculations’ analysis showed no statistically significant differences in both groups before and after the experiment (FPR, *p* = 0.131; CG, *p* = 0.861) (Figure 1).

After the experiment, statistically significant differences were found in all MDS-UPDRS subscales and PDQ-39 in the FPR group. Similar differences, over 20%, were found in the M-EDL (29.15%), nM-DL (25.85%), and MC (23.93%) subscales. In all subscales, a decrease in point values was found, corresponding to the improvement of the studied aspects of the functioning of people with PD. In CG, statistically significant changes were seen in MC, which was associated with an increase in the point value and a substantial deterioration in this area. In the other subscales, the differences were statistically insignificant (Table 3).

Positive changes in the quality of life assessment as measured by PDQ-39. In the FPR group, the score improved significantly, decreasing by −7.50 points, corresponding to a relative improvement in quality of life of 14.31% (*p* < 0.001). In contrast, in CG, the change was statistically insignificant (*p* = 0.899).

## 3. Discussion

Movement therapies can be considered essential supportive methods for treating PD, with a potentially beneficial neuroprotective effect that reduces clinical symptoms and improves quality of life. A literature review provides clear evidence that physical exercise benefits individuals with PD, regardless of the type of exercise [9,36]. However, it is suggested to engage in exercises of greater intensity [37], which may enhance dopaminergic signaling and improve neuroplasticity. The effects of exercise can be explained, among other mechanisms, by synthesizing several brain-derived neurotrophic factors, as presented in systematic reviews with meta-analyses and animal studies [11,14].

The benefits of PA and motor rehabilitation influencing changes in BDNF levels in humans are well-documented, whereas studies involving the precursor molecule (pro-BDNF) are poorly understood [38]. Since proBDNF and mature BDNF exhibit different effects on cell survival and synaptic plasticity [39], we included both parameters in our study before and after a 16-week moderate-intensity FPR program. We observed a statistically significant increase in proBDNF levels.

Although proBDNF may be cleaved intracellularly to release mature BDNF in an activity-dependent manner, it is unclear how efficient this processing is and how much proBDNF is secreted by neurons [40,41]. It is believed that proBDNF plays an essential role in neuroplasticity and neuronal survival. Its increase after FPR may suggest an adaptive response to exercise, potentially enhancing synaptic plasticity and neuroprotection in PD.

Tuon et al. [42] conducted two types of training over 60 days on a rat model of PD and observed an increase in proBDNF levels in the hippocampus and striatum. Their analysis suggested that the exercise-induced rise in proBDNF is more likely related to subsequent BDNF maturation rather than the pro-apoptotic effects of uncleaved proBDNF, as positive behavioral patterns were observed. If cell death via apoptosis had been significantly stimulated by proBDNF, negative consequences for the evaluated parameters would have also been reflected in the behavioral data [42].

In their research, Azevedo et al. [43], investigating the proBDNF level after a single exercise session in individuals with PD, did not observe its increase. However, it is worth noting that their study focused on a one-time physical effort in the early stages of the disease. In our study, participants were in stage III of the Hoehn and Yahr scale. Statistically, significant changes in proBDNF were observed after 16 weeks of regular, targeted, moderate-intensity exercise, which could be attributed to the activation of pathways associated with neurotrophin production [44].

Interestingly, the statistically significant changes in proBDNF were accompanied by an increase in BDNF, although this increase was not statistically significant. While most studies in the literature report an increase in BDNF following physical exercise [10,37,45], new insights into the relationship between PA and BDNF are provided by the findings published by Spartano et al. [46]. They observed no sustained increase in BDNF in middle-aged adults undertaking regular PA. This suggests that the neuroprotective benefits of physical activity are primarily linked to short-term, intense increases in BDNF triggered by individual exercise sessions rather than by maintaining consistently high levels in the resting state [46]. This may also be confirmed by work by Romero Garavito et al. (2025), in which the authors emphasize that the effects of exercise on BDNF levels are based on dynamic, short-term changes initiating neuroplastic pathways, even though the base values of BDNF may not be constantly elevated [47]. A review by Huang et al. (2014) indicates that increases in BDNF after single training sessions are pronounced, while long-term adaptations do not necessarily manifest as elevated levels at rest, which does not rule out the beneficial neuroprotective effects of regular PA [48]. The statistically insignificant changes in BDNF found in our studies may result from its rapid degradation but may be sufficient to activate neuroplastic mechanisms.

Physical exercise is believed to modulate the processing of BDNF, affecting the balance between proBDNF and mature BDNF [49]. The minor increase in mature BDNF relative to proBDNF observed in the studies conducted in this work suggests that converting proBDNF to BDNF may be impaired in individuals with PD. This phenomenon was observed in studies by Piepmeier et al. [50] and Paterno et al. [37], indicating that although exercise may increase proBDNF levels, the processing into BDNF may not be as efficient due to inherent neurodegenerative processes [37,50].

Additionally, the presence of genetic factors, such as the BDNF Val66Met polymorphism, may influence the effectiveness of exercise in increasing BDNF levels. Individuals with the Met allele may show a diminished response to exercise-induced BDNF level increases, further complicating the relationship between exercise intensity and neurotrophic factor levels [10,51].

In addition to pharmacotherapy, physical rehabilitation plays an important role in managing PD symptoms. By influencing changes in neurotrophic factors, it also impacts motor symptoms and the quality of life of individuals with PD. Our research demonstrated that observed changes in concentrations (proBDNF, BDNF) were associated with clinical improvement in individuals with PD, as measured using scales. The conducted studies revealed a statistically significant improvement in scores on the MDS-UPDRS subscales and the PDQ-39 in the exercise group, accompanied by a statistically significant increase in proBDNF and BDNF levels. Since no studies have been published to date that examine proBDNF concerning clinical status and moderate-intensity training, further research is needed to understand the mechanisms regulating proBDNF levels, their impact, and the body’s adaptation to physical exercise.

In the literature, few studies demonstrate a simultaneous increase in BDNF levels and a reduction in the severity of motor symptoms measured by the MDS/UPDRS test as a result of physical exercise [12]. It is unclear whether neurochemical changes caused the improvement in MDS/UPDRS scores, improved motor skills due to dopaminergic medications or physical training, or interactions among any or all of these factors. An example is the study by Angelucci et al. [52], where elevated BDNF levels were observed only after the 7th day of training despite improved motor symptoms and quality of life. The authors suggested that the changes might have been more related to the impact of training on muscle strength rather than plastic changes in the brain. However, it is worth noting that the study was conducted on a small group (nine participants) at stage II of the Hoehn and Yahr scale disease progression.

Azevedo et al. [43] observed a different effect. They implemented a single aerobic exercise session for individuals with PD, after which an increase in BDNF was noted. Still, no changes in proBDNF or correlations with motor symptoms were found. However, this study involved individuals in the early stages of the disease and included only a single exercise session.

The value of our study undoubtedly lies in the large and homogeneous research group (60 individuals) consisting of people in stage III of the disease according to the Hoehn and Yahr scale classification. PA can influence proteolytic processes that transform proBDNF into BDNF, which may improve motor functions and quality of life for individuals with PD [43,53].

## 4. Materials and Methods

### 4.1. Subjects

This study was conducted on people diagnosed with idiopathic PD who were members of Parkinson’s Associations. All participants were informed about the study’s course and expressed their written consent to participate in it. The Bioethics Committee has granted permission to carry out this study. The purposeful sampling technique was applied. A sample size calculator was used to determine the sample size.

Inclusion criteria:

– Diagnosis of Parkinson’s disease based on the United Kingdom Parkinson’s Disease Society Brain Bank criteria stage three of the disease according to the Hoehn and Yahr scale [54];
– Mini-Mental State Examination (MMSE) score of ≥24 [55];
– Beck Depression Inventory (BDI) score < 10, indicating no depression [56];
– No coexisting neurodegenerative diseases;
– A minimum treatment period of 2 years;
– No contraindications for physical exercise.

Exclusion criteria:

– Parkinsonian syndromes other than idiopathic PD;
– Concomitant neurodegenerative diseases;
– Concomitant diseases with reduced exercise tolerance;
– Absence of approval to participate in the study;
– Earlier participation in physical rehabilitation classes.

All participants were under medical supervision. The essential treatment was levodopa, followed by ropinirole, depending on the frequency of use. The levodopa equivalent daily dose (LED) was calculated based on the conversion ratios accepted in the literature review [57]. Any corrections during the study resulted in exclusion from the final analysis.

**Figure 2:**
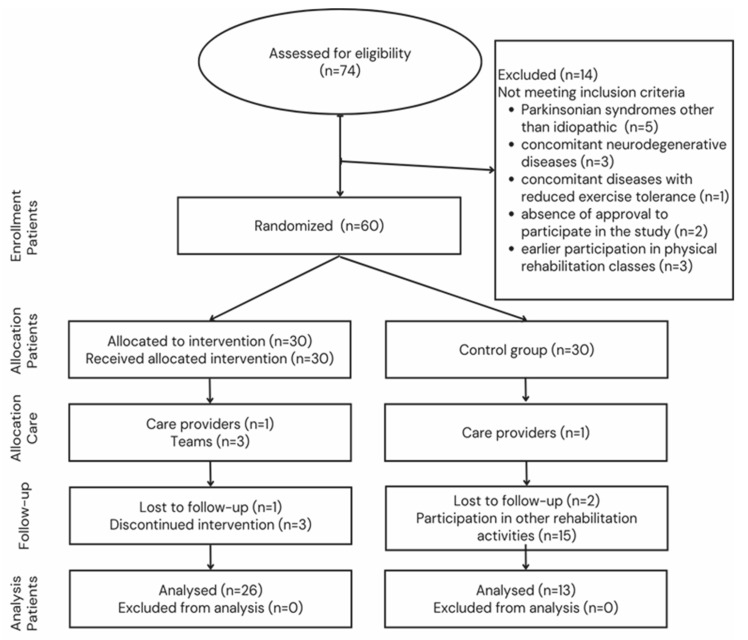
Flowchart of participant selection.

The subjects were randomly assigned to a group of functional physical rehabilitation (FPR) participants (n = 26; age: 63.96 ± 8.88; duration of disease: 7.88 ± 4.59) and the control group (CG) (n = 13; age: 64.08 ± 5.94; duration of disease: 7.5 ± 3.55). Figure 2 shows a detailed flow chart of the participants’ recruitment.

### 4.2. Course of the Experiment (Exercises)

Considering the nature of physical rehabilitation in PD, the lack of definitive rehabilitation standards, the advanced (stage III) progression of the disease, and the specific needs of study participants, people from the exercise group carried out the FPR program. They combined traditional and contemporary approaches with exercises linked to task-based training. The protocol included exercises conducted at specific times and intensities, coordinating movements with stimulating mechanisms, such as visual, auditory, and tactile cues, and mentally visualizing movements before performing them. The instructor focused on identifying postural abnormalities, helping participants recognize these issues, and independently correcting deviations with particular care.

The FPR program included stretching and strengthening exercises, strategies to reduce or halt tremors through intentional movements, and the enhancement of overall fitness, including fine motor skills, balance improvement, conscious shifting of the center of gravity, with special attention to gait improvement, rising from a chair, independent turning while lying down, and enhanced motor coordination in performing simple and complex tasks. All exercises used had a functional rationale, improving the participants’ ability to cope with daily activities.

Participants in the FPR program attended sessions three times per week for 60 min on non-consecutive days for 16 weeks. The sessions were led by a physiotherapist specializing in PD rehabilitation. Each session was divided into three parts: warm-up, main section, and cool-down.

Warm-up: 15 min, consisting of exercises for the cervical spine, flexion-extension movements forward, backward, sideways, and rotations (5 repetitions each direction); torso rotations to the right and left (10 repetitions each side); upper limb exercises with alternating flexion-extension, simultaneous abduction-adduction (10 repetitions per limb); lower limb exercises with support at the side of a chair, flexion and extension of the leg further from the chair, front support on the chair, abduction-adduction (10 repetitions each side); and ankle dorsiflexion and plantarflexion (10 repetitions). Exercises were performed within the maximum individual range of motion as recommended.

Main section: 30 min, divided into 5 intervals (exercises) of 4 min each, performed at 60–70% of maximum heart rate (120–140 beats per minute). Exercises included simulated cycling in a seated position with alternating leg movements; standing push-ups with torso rotation, alternating to the right and left; rising from and sitting on a chair, with alternating lead legs and arm swings; quick walking in a figure-eight pattern between cones marking the path; and rolling from prone to supine with a leg swing, twice to the right and twice to the left.

Each exercise within intervals was performed for 20–30 s, as intensely or quickly as possible without joint impact, followed by a 10–15 s rest before the next repetition. After each interval, a 2-min rest was allowed.

Cooldown: 15 min, consisting of static muscle stretching exercises, as follows: with side support on a chair, pulling the foot of the far leg towards the glutes and holding for 15–20 s (2 repetitions per limb); in a supine position, pulling one knee towards the chest and holding for 15–20 s (2 repetitions per limb); sitting with legs straight, pulling a belt wrapped around the midfoot of one leg and holding for 15–20 s (2 repetitions per limb); and seated on a chair, pulling the elbow of an extended arm towards the head, holding for 15–20 s (2 repetitions per arm).

Exercises were conducted in groups of 10, with each participant equipped with a heart rate monitor. In addition to the lead instructor, trained assistants helped monitor participants’ heart rates and the timing of their efforts.

In line with the Parkinson’s Exercise Guidelines for People with Parkinson’s, safety was the priority. Therefore, during the first three weeks, exercises in the main section were conducted at 40–60% of maximum heart rate, adjusted to individual abilities while maintaining the exercise program’s structure. From the fourth week, the planned intensity was implemented. The FPR program was developed based on literature and the authors’ experience [36,58,59].

The subjects from CG did not participate in any rehabilitation sessions.

### 4.3. Clinical Assessments (Rating Scales)

The MDS UPDRS scale comprises four parts: Part I (Non-Motor Aspects of Daily Living Skills); Part II (Motor Aspects of Daily Living); Part III (Motor Symptoms); and Part IV (Motor Complications), which was used to comprehensively assess the impact of PD on patients’ daily functioning and overall quality of life. Part I consists of two parts: IA, related to numerous behaviors assessed by the investigator based on information received from participants and their caregivers; and IB, which is completed by the patient with or without the aid of the caregiver, irrespective of the investigator. Part II of the MDS-UPDRS scale does not have separate scales to assess ON and OFF states. Part III contains instructions for the investigator to submit to the patient, and the investigator completes it. Part IV consists of instructions for the investigator that should be read to the patient. This section combines information from the patient with information from clinical inspection and the investigator’s opinion. Each subscale has 0–4 ratings, where 0 = normal, 1 = slight, 2 = mild, 3 = moderate, and 4 = severe [60,61].

To assess the quality of life of the participants, the Parkinson’s Disease Questionnaire—PDQ-39 was used [62]. The PDQ-39 scale consists of 39 questions organized into 8 subscales: mobility, activities of daily living, emotional well-being, stigma, social support, cognitive function, communication, and bodily discomfort. The scoring system is a 5-point scale ranging from 0 to 4 (0—never; 1—rarely; 2—sometimes; 3—often; 4—always). The questions refer to the past month and are closely related to the presence of PD. Participants filled out the questionnaire by hand. A separate score was calculated for each subscale. The overall questionnaire score was presented as the Summary Index (SI), calculated using the formula: PDQ-SI = total score/8. The maximum possible score for each subscale was 100, indicating the poorest quality of life for the patient.

### 4.4. Blood Samples Collection

Blood samples were collected between 8:00 and 10:00 a.m. after an overnight fast the day after the final rehabilitation session. Blood (4 mL) was taken from the forearm vein into EDTA-2Na-containing vacuum tubes (BD Vacutainer, Becton, Dickinson and Company, Franklin Lakes, NJ, USA) and centrifuged at 3000× *g* for 15 min at 4 °C. The supernatant serum was collected and stored at −80 °C until used for analyses.

Measurements of proBDNF and BDNF levels by enzyme-linked immunosorbent assays (ELISA). The following two-site sandwich ELISA kits were used: the human BDNF (ChemiKine, Merck Millipore, Burlington, MA, USA) and the human proBDNF (Wuhan Fine Biotech Co., Ltd., Wuhan, China). Serum samples obtained to determine proBDNF and BDNF were kept at −80 °C until the analyses. On the day of the assays, the samples were centrifuged once more (2500× *g*, 10 min, 4 °C), and the assays were performed according to the manufacturer’s protocol. The absorbance was measured at 450 nm using a multi-well reader (ELx800, BioTek Instrumente, Inc., Winooski, VT, USA). For the proBDNF assay, the intra-assay precision coefficient of variation was <8%, and the detection limit was <0.094 ng/mL. For the BDNF assay, the intra-assay coefficient of variation was 3.7%, and the detection limit was 7.8 pg/mL.

### 4.5. Statistical Analysis

Descriptive statistics methods were used to present the results obtained on a quantitative scale. Basic statistical measures were calculated: arithmetic mean (M), median (Mdn), standard deviation (SD), interquartile range (IQR), minimum (Min), and maximum (Max). The Shapiro–Wilk test was used to assess the normality.

The analysis of variance (ANOVA) with repeated measures was used to determine the significance of differences in mean values for results consistent with the normal distribution. The Bonferroni test was used as a post hoc test. The Wilcoxon signed-rank test was used to determine differences between two measurements without a normal distribution. In contrast, the Mann–Whitney test determined the difference between groups. Results at *p* < 0.05 were considered statistically significant. Statistical analysis was performed using Statistica v.13.0 (StatSoft, Tulsa, OK, USA).

## 5. Conclusions

The research found that a 16-week moderate-intensity FRP in individuals with PD significantly increased the level of proBDNF, with a slight increase in BDNF, which may indicate an impaired conversion process. However, the improvement in clinical conditions and quality of life may suggest that these changes are related to the later maturation of BDNF.

Our findings are significant as they underscore the need to explore methods to enhance the benefits obtained through rehabilitation, especially considering a longer rehabilitation duration. An increase in BDNF levels could lead to greater neuroplasticity and facilitate improvements in motor function, while proBDNF, as a precursor, may stimulate its development.

## Figures and Tables

**Table 1:** Characteristics of subjects.

Characteristic		Group FPR (n = 26)	Group CG (n = 13)	t-Student Test	
		M ± SD	M ± SD	T	*p*
Age (years)		63.96 ± 8.88	64.08 ± 5.94	0.042	0.967
Disease duration (years)		7.88 ± 4.59	7.05 ± 3.55	0.238	0.813
MDS-UPDRS (pts)	nM-DL	13.54 ± 2.89	13.38 ± 4.48	0.130	0.897
	M-EDL	17.15 ± 3.69	17.00 ± 2.55	0.135	0.894
	ME	33.46 ± 5,22	33.77 ± 5.46	−0.171	0.865
	MC	8.19 ± 2.23	8.23 ± 1.23	−0.058	0.954
PDQ-39 (pts)		52.42 ± 8.00	52.08 ± 7.84	0.128	0.899

FPR—functional physical rehabilitation group; CG—control group; MDS-UPDRS—movement disorder society-sponsored revision of the Unified Parkinson’s Disease Rating Scale, nM-DL—non-motor aspects of daily living skills (part I of MDS-UPDRS); M-EDL—motor experiences of daily living (part II of MDS-UPDRS); ME—motor examination (part III of MDS-UPDRS); MC—motor complications (part IV of MDS-UPDRS); PDQ-39—Parkinson’s Disease Questionnaire; M—arithmetic mean; SD—standard deviation; T—value of T-test; *p*—probability degree.

**Table 2:** The concentration of proBDNF and BDNF in the study groups before and after the experiment.

	FPR Group		*p*	CG Group		*p*
	Before	After		Before	After	
proBDNF (ng/mL)	11.67 ± 3.92	16.27 ± 6.53	0.006 *	11.68 ± 2.23	12.86 ± 4.43	1.0
BDNF (pg/mL)	20,391.36 ± 6550.00	22,818.87 ± 5341.59	0.131	20,518.57 ± 6391.70	20,362.39 ± 2525.74	0.861

FPR—functional physical rehabilitation; CG—control group; *p*—probability degree; *—statistically significant differences.

**Table 3:** Comparison of the results of the tests used before and after the experiment.

		FPR			CG		
		Absolute Difference II—I (pts)	Relative Difference Δ (%) II—I	*p*	Absolute Difference II—I (pts)	Relative Difference Δ (%) II—I	*p*
MDS-UPDRS	nM-DL (F = 59,243, *p* = 0.00)	−3.5	−25.85	*p* < 0.01 *	−0.07	−0.52	*p* < 1
	M-DL (F = 34,844, *p* = 0.00)	−5.0	−29.15	*p* < 0.001 *	0.77	4.53	*p* < 1
	ME (F = 39,585, *p* = 0.00)	−0.62	−1.82	*p* < 0.001 *	1.23	3.64	*p* < 1
	MC (F = 11,243, *p* = 0.00)	−1.96	−23.93	*p* < 0.001 *	0.77	9.36	*p* < 0.001
PDQ-39 (F = 81.078, *p* = 0.000)		−7.50	−14.31	*p* < 0.001 *	1.15	2.21	*p* < 0.899

I—before experiment; II—after experiment; *p*—probability degree; *—statistically significant differences.

## Data Availability

Data are contained within the article.
